# Serum interleukin-6, procalcitonin, and C-reactive protein at hospital admission can identify patients at low risk for severe COVID-19 progression

**DOI:** 10.3389/fmicb.2023.1256210

**Published:** 2023-10-23

**Authors:** Christian Matthias Zobel, Werner Wenzel, Jan Philipp Krüger, Ulrich Baumgarten, Tobias Wagelöhner, Nino Neumann, Behruz Foroutan, Rico Müller, Annette Müller, Dominic Rauschning, Meike Schüßler, Lorenz Scheit, Felix Weinreich, Klaas Oltmanns, Franziska Keidel, Maria Koch, Sebastian Spethmann, Maximilian Schreiner

**Affiliations:** ^1^Department of Internal Medicine, Bundeswehr Hospital, Berlin, Germany; ^2^Department of Microbiology and Hospital Hygiene, Bundeswehr Hospital, Berlin, Germany; ^3^Department of Laboratory Medicine, Bundeswehr Hospital, Berlin, Germany; ^4^Department of Internal Medicine, Bundeswehr Central Hospital, Koblenz, Germany; ^5^Department of Internal Medicine, Bundeswehr Hospital, Hamburg, Germany; ^6^Department of Internal Medicine, Bundeswehr Hospital, Westerstede, Germany; ^7^Department of Cardiology, Angiology and Intensive Care Medicine|CCM, Deutsches Herzzentrum der Charité – Medical Heart Center of Charité and German Heart Institute Berlin, Berlin, Germany; ^8^Charité – Universitätsmedizin Berlin, Corporate Member of Freie Universität Berlin and Humboldt-Universität zu Berlin, Berlin, Germany

**Keywords:** SARS-CoV-2, neopterin, ARDS, cytokine release syndrome, mechanical ventilation

## Abstract

**Background:**

COVID-19 can show a variable course, from asymptomatic infections to acute respiratory failure and death. For efficient allocation of resources, patients should be stratified according to their risk for a severe course as early as possible.

**Methods:**

135 hospitalized patients with COVID-19 pneumonia at four German hospitals were prospectively included in this observational study. A standardized clinical laboratory profile was taken at hospital admission and a panel of serum markers with possible roles in the COVID-associated cytokine storm were also determined. 112 patients could be evaluated. The primary endpoint of ventilator requirement or death within 30 days of symptom onset was met by 13 patients.

**Results:**

Serum elevations of interleukin-6 (IL-6), procalcitonin (PCT), and C-reactive protein (CRP) at hospital admission were each highly significantly (*p* < 0.001) associated with ventilator requirement/death within 30 days of symptom onset. With a sensitivity of 92% and a specificity of 65–67%, IL-6 ≥ 52.8 pg/ml, PCT ≥ 0.11 ng/ml, and CRP ≥ 71.1 mg/L were predictive of a severe course of COVID-19. Positive likelihood ratios were between 2.6–2.8 and negative likelihood ratios were between 0.11–0.13 for these three markers.

**Conclusion:**

Negative likelihood ratios indicate that IL-6, PCT, and CRP at hospital admission can be used for identifying patients at low risk for severe COVID-19 progression.

## Highlights

- IL-6, PCT, and CRP at hospital admission can identify patients at low risk for severe COVID-19 progression.
- Neopterin is not an independent predictor of severe COVID-19 progression.
- Other serum markers with a potential role in the COVID-associated cytokine storm were not predictive of severe COVID-19 progression in routine clinical practice.

## 1. Introduction

COVID-19 is a viral infection caused by SARS-CoV-2, which emerged from Wuhan, China, in 2019 and spread pandemically within a very short time. It causes relevant morbidity and mortality (Huang et al., 2020). Healthcare systems worldwide have faced immense challenges – medically, in terms of human resources, and economically. Even in industrialized nations with highly developed health care systems, excess mortality still occurs today (Collaborators, 2022).

Clinical manifestations are heterogeneous, ranging from asymptomatic infections to acute respiratory distress syndrome (ARDS). Severe manifestations requiring intensive care occurred in up to 11% of cases and were then associated with mortality of up to 50%, attributable to ARDS and multiorgan failure (Li J. et al., 2021). Patients affected by COVID-19 are often only mildly compromised in the early phase of their infection, but their condition can rapidly deteriorate within a very short time. Being able to assess the severity of the disease as early as possible in the clinical course is therefore of paramount importance in order to make efficient use of limited resources such as inpatient beds or mechanical ventilators.

Several COVID-19-typical changes are visible in the blood count: COVID-19 infection is associated with a decreased number of lymphocytes and eosinophil granulocytes in the vast majority of patients. Leukocytosis, neutrophilia, and thrombocytopenia are associated with poor outcome. In clinical chemistry, C-reactive protein (CRP), procalcitonin (PCT), interleukin-6 (IL-6), and ferritin are known biomarkers that are elevated in the setting of acute inflammation and may help predict an unfavorable outcome in COVID-19. Other laboratory parameters that may be elevated in severe COVID-19 include D-dimers as markers of coagulation activation and cardiac enzymes such as creatine kinase (CK) and troponin I [for a review of COVID-19 related laboratory changes, see: Ponti et al. (2020)].

The biomarker neopterin recently experienced a renaissance in the context of the COVID-19 pandemic. It is produced by macrophages after interferon-gamma stimulation and represents a correlate for the activation of type 1 T helper cells. Neopterin can indicate the activity of various viral diseases such as HIV or SARS-CoV-1 (Murr et al., 2002; Zheng et al., 2005). Serum neopterin concentrations were higher in COVID-19 infected patients than in a healthy comparison group. Furthermore, higher neopterin concentrations were found in severely ill patients with COVID-19 than in mildly ill patients (Robertson et al., 2020; Bellmann-Weiler et al., 2021; Chauvin et al., 2021; Ozger et al., 2021; Karacaer et al., 2022).

Early in the pandemic, dysregulation of inflammatory cells with a release of proinflammatory cytokines was observed in a group of patients with a severe course of COVID-19 and was soon described as a “cytokine storm.” This is related to cytokine release syndrome and other hyperinflammatory syndromes such as hemophagocytic lymphohistiocytosis (Del Valle et al., 2020; Mehta et al., 2020; Moore and June, 2020; Li X. et al., 2021). These syndromes are associated with significant dysregulation of pro- and anti-inflammatory cytokines and are characterized by a very poor prognosis (Ramos-Casals et al., 2014).

This study aimed to identify biomarkers that provide an early indication of the likelihood of ventilator requirement or death in hospitalized COVID-19 patients. For this purpose, we evaluated a panel of routine clinical laboratory parameters relevant for diagnosis, progression, and complications of COVID-19 disease. Furthermore, we screened a selection of cytokines, chemokines, growth factors, and other markers that may play a role in the COVID-19 associated cytokine storm for their diagnostic potential.

## 2. Methods

### 2.1. Study design and participants

Patients were recruited for this prospective observational study (Predictive Biomarkers for Severe Progression of COVID-19 – PreBiSeCov) at the Bundeswehr Hospitals in Berlin, Hamburg and Westerstede and the Bundeswehr Central Hospital Koblenz. We aimed to detect early predictive factors for severe disease progression in the blood of hospitalized COVID-19 patients. The study was conducted in accordance with the principles of the Declaration of Helsinki (World Medical Association, 2013) and approved by the Ethics Committee of the Berlin Medical Association (file number Eth-10/20). All patients gave written informed consent for study participation.

The study protocol was published in the German Clinical Trials Registry prior to inclusion of the first patient (ID: DRKS00021591).^1^ Adult, immunocompetent patients with clinical suspicion of COVID-19 pneumonia and indication for hospital admission were included. Due to the clinical inclusion criterion, patients could be excluded retrospectively according to the study protocol if they tested negative for SARS-CoV-2 twice by PCR during the inpatient course and also had no signs of COVID-19 on computed tomography of the chest.

Blood was drawn from the patients at the following time points: within 24 h of hospital admission as well as 7, 10, 14, and 21 days from symptom onset. Blood was drawn only during the inpatient stay, i.e., if a patient was admitted after or discharged before any of the time points from symptom onset, these data are missing. The blood was centrifuged, the serum was pipetted off and stored at −20°C for later analysis. Vital signs were obtained at the above time points using standardized study forms, and the following parameters from the local hospital laboratory were recorded: Hemoglobin, platelets, leukocytes, automated eosinophil and lymphocyte counts, PCT, CRP, ferritin, creatinine, urea, sodium, potassium, alanine aminotransferase (ALAT), aspartate aminotransferase (ASAT), gamma-glutamyltransferase (gamma-GT), alkaline phosphatase (AP), bilirubin, lactate dehydrogenase (LDH), prothrombin time (PT) ratio, partial thromboplastin time (PTT), D-dimers, CK, CK-MB, NT-proBNP, troponin T.

The primary endpoint was requirement of non-invasive or invasive ventilation within 30 days of symptom onset, which was assessed using medical records. For the purpose of this study, high-flow nasal cannula (HFNC) was also considered as non-invasive ventilation. While HFNC is usually considered different from non-invasive ventilation, it has similar effects by providing continuous positive airway pressure and a higher fraction of inspired oxygen (up to 100%) compared to a simple face mask or nasal cannula (Renda et al., 2018). Furthermore, the benefits of HFNC in COVID-19 were not immediately clear at the beginning of the pandemic (which coincides with the beginning of this study), which might have led to ventilation therapy that might be considered unnecessary in hindsight. Also, due to supply constraints, HFNC was only available towards the end of the study period in all participating hospitals. Therefore, we felt that excluding HFNC from the “severe” group of patients would skew the data. This decision affected three patients who only received HFNC and no other ventilation therapy.

In deviation from the study protocol, death within 30 days of symptom onset was additionally considered to meet the primary endpoint. This specific change affected only one patient who had an indication for ventilation but refused it and subsequently died of COVID-19 without prior ventilation. Reaching the primary endpoint is considered a “severe course” of COVID-19.

The secondary endpoint was death from any cause within 1 year of symptom onset. Here, telephone numbers from the medical records were used to try to reach patients or their relatives.

### 2.2. Measurement of serum biomarkers

The following ELISA kits were used to measure biomarkers in serum: Human CTGF, Human FGF-basic (bFGF), Human IL-12, Human TIMP-1, Human MCP-1 (CCL-2), Human RANTES (CCL-5), Human IL-10 (all: Standard ABTS ELISA Development Kits from Peprotech, London, UK); Human CXCL-2/GROβ, Human CXCL-5/ENA-78, Human IL-1α/IL-1F1, Human IL-1β/IL-1F2, Human IL-2, Human IL-8/CXCL8, Human MMP-9, Human TGF-β1, Human IFN-β, Human IFN-γ (all: DuoSet ELISA Development Systems from R&D Systems, Minneapolis, MN, USA); Human IL-4 Uncoated ELISA (Invitrogen/Thermo Fisher Scientific, Waltham, MA USA). TNF-α and IFN-α could not be measured as originally planned due to supply constraints. Instead, deviating from the study protocol, neopterin was additionally determined by ELISA (IBL International, Hamburg, Germany). All measurements except IL-6 were automated on a Euroimmun Analyzer I (Euroimmun, Lübeck, Germany). IL-6 was measured on a Cobas 6,000 analyzer system using the Elecsys immunoassay (both: Roche Diagnostics, Mannheim, Germany).

In the sera of the first 35 patients all above mentioned biomarkers were determined. We found that the following factors had insufficient reproducibility, due to loss of stability or low sensitivity of test kits: CXCL-2, CXCL-5, IL-1α, IL-1β, IL-2, IL-8, MMP-9, TGF-β1, IFN-β, IFN-γ, IL-4 (data not shown). Therefore, these proteins were not further measured or evaluated.

### 2.3. Statistical analysis

Following a practical approach to clinical care, only the parameters measured in the earliest blood draw, i.e., within 24 h of inpatient admission, were used to calculate a correlation with the primary endpoint. Correlations were calculated using the Mann–Whitney U test. Effect sizes for the Mann–Whitney U test were calculated using Pearson’s r (Fritz et al., 2012). *p*-values were corrected for multiple testing using the Benjamini-Hochberg procedure (false discovery rate; Benjamini and Hochberg, 1995). Two-tailed *p*-values < 0.05 were considered statistically significant.

Furthermore, the parameters were tested for multicollinearity using Pearson’s product–moment correlation, with correlation values above 0.8 considered indicative of multicollinearity.

For categorical data, Fisher’s exact test was preferred over the chi-square test due to small sample sizes.

Since only less than half of the patients could be reached for telephone follow-up 1 year after inclusion of the last patient, the secondary end point was not evaluated.

Statistical analyses were performed with SPSS 28.0.1 (IBM Corp.) and the STATS PADJUST 1.04 extension after consultation with a biostatistician. Graphical analyses of the data were performed with GraphPad Prism 9.3.1 (GraphPad Software, Inc.).

## 3. Results

### 3.1. Patient characteristics

**Figure 1:**
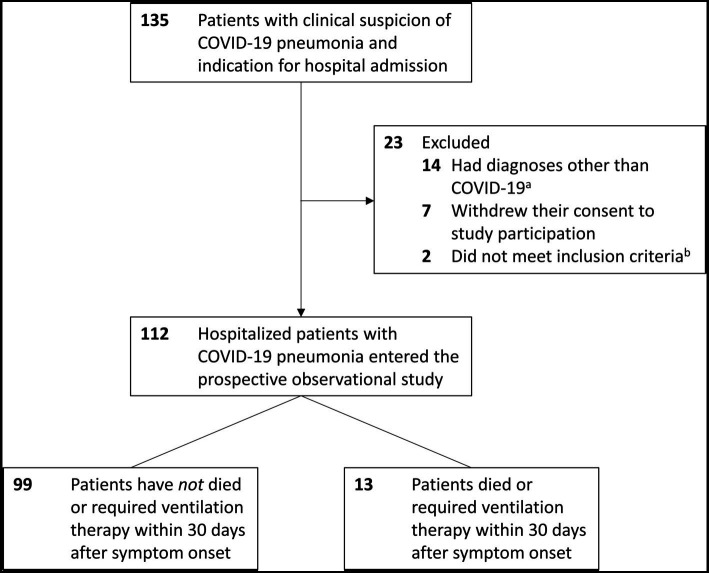
Flow of participants. ^a^Bacterial pneumonia (5 patients), asthma, Pneumocystis pneumonia (2 patients each), newly diagnosed lung cancer, decompensated heart failure, acute pulmonary embolism, radiation pneumonitis, psychosomatic complaints (1 patient each). ^b^Both patients were on immunosuppressive therapy, which was not known at the time of hospital admission.

Between 29 April 2020 and 15 December 2020, a total of 135 patients with clinical suspicion of COVID-19 pneumonia and indication for hospital admission were included in the study. Of these, 23 patients were subsequently excluded: 14 had diagnoses other than COVID-19 and were excluded according to the previously established study protocol (twice negative SARS-CoV-2 PCR and no signs of viral pneumonia on computed tomography of the thorax), seven patients withdrew their consent to study participation, and two patients with the exclusion criterion “immunosuppression” were included by mistake. Thus, 112 patients remained in the study, all of whom had at least one nasopharyngeal swab with a positive SARS-CoV-2 PCR result. Of these, 13 patients met the primary endpoint of “death or ventilation within 30 days of symptom onset” (Figure 1).

The two groups showed differences in their demographic and clinical characteristics at inclusion (Table 1). Patients with outcome of death/ventilation were older (72 vs. 59 years), predominantly male (92 vs. 63%), and more often had preexisting conditions. Therapy of patients with poor outcome more often included corticosteroids (77 vs. 35%) and antibiotics (70 vs. 56%). Furthermore, their initial oxygen saturation was lower (sO_2_ 89 vs. 95%). There were no differences in duration between symptom onset and hospital admission (both 7 days). The body mass index of patients with poor outcome tended to be lower (25.22 vs. 29.2).

**Table 1:** Baseline characteristics of study participants.

	No ventilation (*n* = 99)	Ventilation/death (*n* = 13)
Age, median (range), years	59 (24–91)	72 (42–84)
Sex, No. (%)		
Men	62 (63)	12 (92)
Women	37 (37)	1 (8)
BMI^a^, median (IQR)	29.20 (24.95–33.55) [*n* = 74]	25.22 (23.00–34.14) [*n* = 7]
Interval between symptom onset and hospital admission, median (IQR), days	7 (4–9)	7 (2.5–9.5)
Radiological signs of (atypical) pneumonia on chest radiograph or CT within 48 h of hospital admission^a^, No. (%)	87 (88)	13 (100)
No. of patients with CT of the chest^a^ (%)	83 (84)	11 (85)
Oxygen supplementation at hospital admission, No. (%)	41 (41)	11 (85)
Vital signs at hospital admission, median (IQR)		
SpO_2_, %	95 (91–97)	89 (84.5–91.5)
Systolic BP, mmHg	129 (118–140)	130 (107–149)
Diastolic BP, mmHg	78 (70–82)	80 (71.5–90)
Heart rate, min^−1^	80 (74–92)	86 (72.5–98)
Respiratory rate, min^−1^	16 (14–20)	18 (16–20)
Previous illnesses, No. (%)		
Arterial hypertension	51 (52)	10 (77)
Treatment with ACE inhibitor or ARB	42 (42)	9 (69)
Cardiovascular disease (except arterial hypertension)	30 (30)	10 (77)
Diabetes mellitus	25 (25)	8 (62)
Active cancer	1 (1)	0 (0)
Lung disease	7 (7)	3 (23)
Liver disease	8 (8)	0 (0)
Chronic kidney disease	13 (13)	4 (31)
Other diseases	53 (54)	10 (77)
Therapy, No. (%)		
Dexamethasone or prednisolone	35 (35)	10 (77)
Remdesivir	32 (32)	5 (38)
Antibiotic therapy^a^	31 (56) [*n* = 55]	7 (70) [*n* = 10]
Prevalence of positive bacterial culture (all locations)^a^, No. (%)	7 (7)	2 (15)

IQR, interquartile range; BMI, body mass index; CT, computed tomography; BP, blood pressure; ARB, Angiotensin II receptor blocker.

a These items were not part of the previously planned data collection and were added during the course of the study or collected retrospectively; therefore, data is missing for some participants.

Since no COVID-19 vaccines were authorized in Germany at the time of the study, all patients were unvaccinated.

### 3.2. Early prediction of severe COVID-19 by serum markers

The following nine biomarkers were determined in the sera of all 112 patients at hospital admission: CTGF, bFGF, IL-12, TIMP-1, CCL-2, CCL-5, IL-10, IL-6, and neopterin. In addition, 25 laboratory parameters from local hospital laboratories (see 2.1) were evaluated. Thus, a total of 34 parameters were evaluated for their potential to predict severe COVID-19 progression. Eleven parameters were significantly associated with the outcome of death/ventilator requirement within 30 days of symptom onset. Medians and significance levels of serum markers for the two groups are summarized in Table 2.

**Table 2:** Median values and significance levels of serum markers at hospital admission of patients with COVID-19 pneumonia.

	Median (95% confidence interval)			
Serum marker	No ventilation	Ventilation/death	*p*-value	Adjusted *p*-value
IL-6	32.35 pg/ml (22.6–48.4) [*n* = 96]	126.7 pg/ml (75.3–202.7) [*n* = 12]	<0.0001	<0.001^***^
PCT	0.08 ng/ml (0.06–0.09) [*n* = 97]	0.25 ng/ml (0.12–0.93) [*n* = 13]	<0.0001	<0.001^***^
CRP	48.1 mg/L (35.2–66) [*n* = 99]	139 mg/L (74–225.1) [*n* = 13]	<0.0001	<0.001***
Troponin T	8.0 ng/L (5.0–10) [*n* = 93]	24 ng/L (8.0–37) [*n* = 13]	0.002	0.014*
Urea	28.4 mg/dl (25.5–32) [*n* = 70]	49.4 mg/dl (33–127.1) [*n* = 10]	0.004	0.024*
NT-proBNP	88 pg/ml (63.4–139.2) [*n* = 91]	475.1 pg/ml (113.8–1,625) [*n* = 12]	0.004	0.024*
Neopterin	20.44 nmoL/L (17.67–24.81) [*n* = 95]	41.34 nmoL/L (22.11–68.36) [*n* = 13]	0.007	0.031*
Lymphocyte count	1.05/nl (0.9–1.2) [*n* = 96]	0.75/nl (0.67–1.03) [*n* = 13]	0.007	0.031*
LDH	286 U/L (255–305) [*n* = 98]	383.5 U/L (286–434) [*n* = 12]	0.008	0.031*
Creatinine	0.92 mg/dl (0.9–1.0) [*n* = 97]	1.27 mg/dl (0.87–2.48) [*n* = 13]	0.010	0.034*
ASAT	36 U/L (31–40) [*n* = 97]	47 U/L (37–77) [*n* = 12]	0.016	0.048*
Sodium	137 mmol/L (137–138) [*n* = 94]	134.5 mmol/L (130–137) [*n* = 12]	0.025	0.067
CK-MB	17 U/L (15–19) [*n* = 91]	22 U/L (16–29) [n = 13]	0.026	0.067
PTT	29.8 s (28.2–31.7) [*n* = 95]	33.3 s (31.6–37.3) [*n* = 12]	0.032	0.077
Hemoglobin	13.6 g/dl (13.2–14.1) [*n* = 99]	12.4 g/dl (11.6–13.4) [*n* = 13]	0.035	0.079
Eosinophil count	0.01/nl (0.01–0.02) [*n* = 96]	0/nl (0–0.02) [*n* = 13]	0.060	0.120
CK	99 U/L (82–112) [*n* = 94]	156 U/L (74–862) [*n* = 13]	0.058	0.120
Ferritin	400 μg/L (304–642) [*n* = 87]	850.8 μg/L (252.4–1868) [*n* = 13]	0.088	0.166
Leukocytes	5.57/nl (5.0–6.1) [*n* = 99]	6.62/nl (3.35–9.58) [*n* = 13]	0.121	0.217
Platelets	213.5/nl (198–226) [*n* = 98]	173/nl (144–252) [*n* = 13]	0.133	0.225
IL-10	496.3 pg/ml (388.7–719.8) [*n* = 91]	390.2 pg/ml (83.22–989.9) [*n* = 13]	0.240	0.340
Potassium	4.075 mmol/L (4.0–4.2) [*n* = 92]	4.3 mmol/L (3.52–5.52) [*n* = 11]	0.236	0.340
Bilirubin	0.5 mg/dl (0.42–0.59) [*n* = 80]	0.485 mg/dl (0.4–1.1) [*n* = 10]	0.220	0.340
D-Dimers	0.621 mg/L (0.48–0.85) [*n* = 87]	0.855 mg/L (0.34–1.685) [*n* = 11]	0.233	0.340
bFGF	361.6 pg/ml (320.6–460.5) [*n* = 95]	539 pg/ml (164.4–1,622) [*n* = 12]	0.252	0.343
PT ratio	101.5% (97–108) [*n* = 96]	96% (72–110) [*n* = 12]	0.335	0.439
IL-12	401.1 pg/ml (320.1–503.2) [*n* = 87]	531.4 pg/ml (202.6–6,108) [*n* = 13]	0.433	0.545
TIMP-1	10,845 pg/ml (9806–15,435) [*n* = 95]	14,527 pg/ml (4511–30,698) [*n* = 13]	0.530	0.644
CCL-5	5,093 pg/ml (1942–7,238) [*n* = 93]	1716 pg/ml (691–10,350) [*n* = 13]	0.586	0.664
CTGF	1,089 pg/ml (805.7–1,438) [*n* = 90]	1,346 pg/ml (372.8–4,538) [*n* = 13]	0.606	0.664
AP	65 U/L (59–70) [*n* = 86]	71 U/L (36–92) [*n* = 9]	0.598	0.664
ALAT	27.5 U/L (24–33) [*n* = 98]	27 U/L (17–46) [*n* = 13]	0.673	0.715
Gamma-GT	38 U/L (34–46) [*n* = 89]	41.5 U/L (20–88) [*n* = 10]	0.697	0.718
CCL-2	195.2 pg/ml (153.5–238.6) [*n* = 91]	203.3 pg/ml (109.9–384.4) [*n* = 13]	0.810	0.810

**Figure 2:**
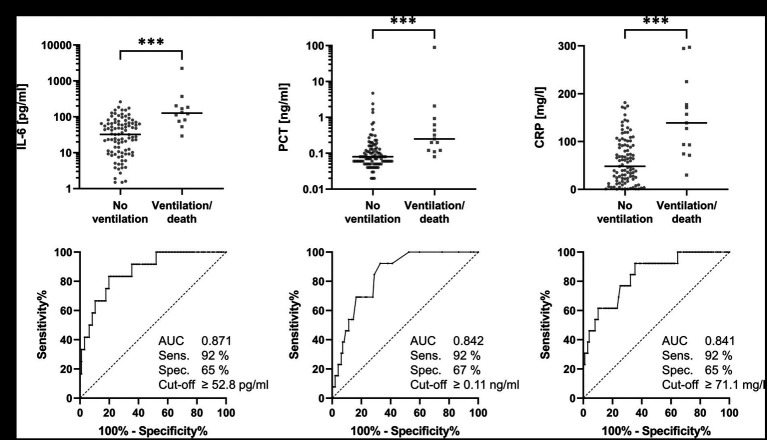
IL-6, PCT, and CRP at hospital admission of patients with COVID-19 pneumonia. Concentrations and receiver operating characteristic curves for IL-6 **(A)**, PCT **(B)**, and CRP **(C)** at hospital admission for outcome prediction (no ventilation vs. ventilation/death) 30 days after symptom onset of COVID-19 infection. The horizontal bars in the upper half of the figure represent medians. All three parameters show similar sensitivity and specificity for predicting severe COVID-19 progression.

Elevation of each of the three inflammatory parameters IL-6, PCT, and CRP was highly significantly (*p* < 0.001) associated with death/ventilator requirement within 30 days of symptom onset (Figure 2). Effect sizes (Pearson’s r) were 0.402 (IL-6), 0.382 (PCT), and 0.376 (CRP), indicating a moderate effect for these markers. Diagnostic accuracy was highest for IL-6 with an area under the curve (AUC) of 0.871 with a 95% confidence interval (CI) of 0.774–0.967, followed by PCT with 0.842 (95% CI 0.754–0.931) and CRP with 0.841 (95% CI 0.731–0.950). Assuming a sensitivity of at least 90%, all three parameters showed similarly high specificity: IL-6 had a specificity of 65% at a cut-off value ≥52.8 pg/ml, PCT had a specificity of 67% at a cut-off value ≥0.11 ng/ml, and CRP had a specificity of 65% at a cut-off value ≥71.1 mg/L. The positive likelihood ratios for IL-6, PCT, and CRP above these cut-off values were 2.6, 2.8, and 2.6, respectively. The negative likelihood ratios for IL-6, PCT, and CRP below these cut-off values were 0.13, 0.11, and 0.12, respectively.

As these inflammatory markers are influenced by bacterial (co-)infection, we performed a retrospective analysis of positive bacterial cultures during hospital admission. We found 9 patients with positive bacterial cultures in our cohort: 6 patients had urinary tract infections, one patient had viridans streptococci in bronchoalveolar lavage fluids, one patient had a staphylococcal bloodstream infection, and one patient had both positive bloodstream culture (*S. aureus*) and urinary culture. We repeated our analyses after exclusion of these patients but found only minor changes. The medians of IL-6, PCT, and CRP decreased slightly for both groups but changes were still highly significant (Supplementary Table 1). Effect sizes were also slightly decreased but still showed a moderate effect, and the AUC was virtually unchanged (Supplementary Table 2).

A score was developed using the above mentioned cut-off values for IL-6, PCT, and CRP (Table 3). Patients who presented upon admission with all three markers below these cut-off values (i.e., a score of 0) were not at risk for ventilation or death.

**Table 3:** A combined score of IL-6, PCT, and CRP.

Score	No ventilation (*n* = 94)	Ventilation/death (*n* = 12)
0	46	0
1	19	1
2	19	4
3	10	7
0	46	0
≥1	48	12

106 patients had complete data of all three markers (IL-6, PCT, and CRP). For every marker above its cut-off value (IL-6 ≥ 52.8 pg/ml, PCT ≥ 0.11 ng/ml, and CRP ≥ 71.1 mg/L), a score of one was added. Hence, every patient was assigned a score ranging from 0 to 3. A score of ≥1 was highly significantly associated (*p* = 0.001) with ventilation or death within 30 days of symptom onset.

Decreased lymphocyte counts (AUC 0.733; 95% CI 0.615–0.852) on admission were also significantly associated with a poor outcome of COVID-19, as were elevations of creatinine (AUC 0.721; 95% CI 0.549–0.893), urea (AUC 0.781; 95% CI 0.596–0.965), troponin T (AUC 0.769; 95% CI 0.645–0.893), NT-proBNP (AUC 0.755; 95% CI 0.623–0.886), LDH (AUC 0.734; 95% CI 0.583–0.885), ASAT (AUC 0.714; 95% CI 0.569–0.860), and neopterin (AUC 0.730; 95% CI 0.585–0.874).

### 3.3. Correlation of neopterin with renal function

Because the size of the smaller group comprised only nine to 13 patients, depending on the parameter, establishing a logistic regression model with multiple variables to predict severe disease progression was not reasonable (Peduzzi et al., 1996). Nevertheless, as preliminary work toward a logistic regression model, all parameters were tested for multicollinearity using bivariate correlation. The Pearson correlation matrix for all parameters can be found in Supplementary Table 3.

**Figure 3:**
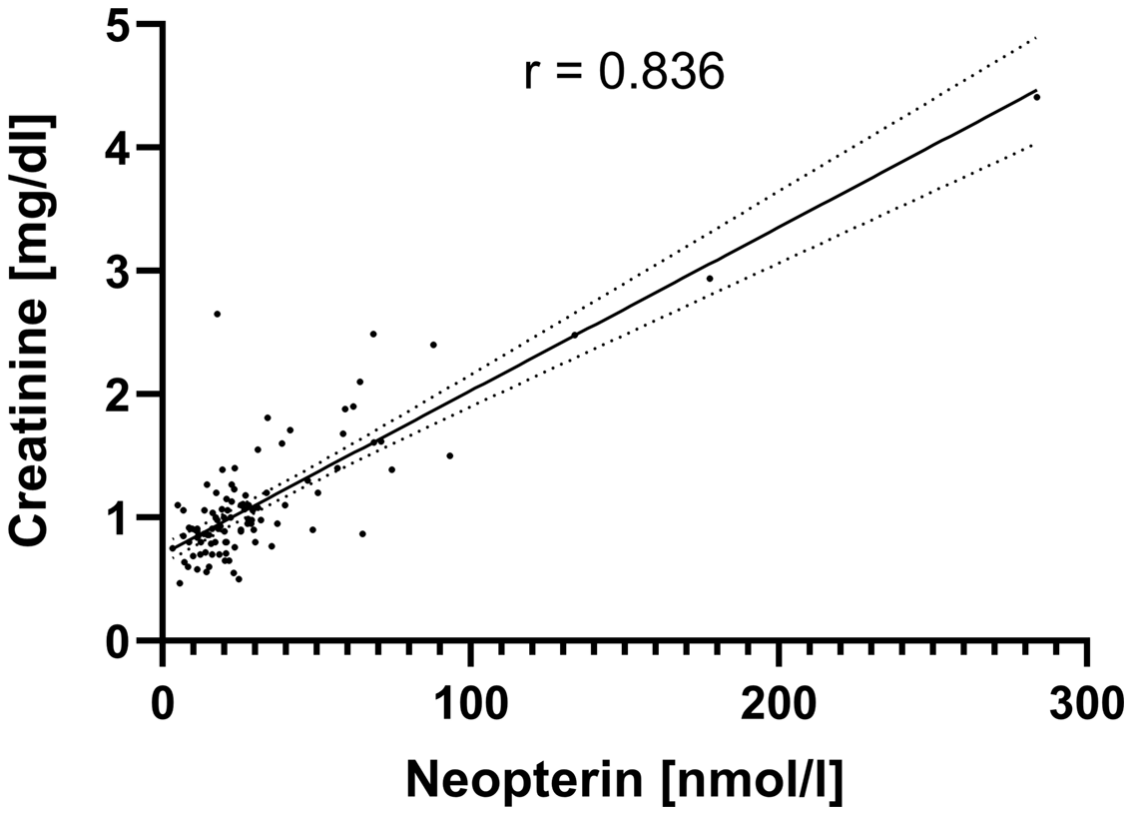
Correlation between creatinine and neopterin in hospitalized patients with COVID-19 pneumonia. The scatter plot with linear regression line and 95% confidence interval shows a strong correlation (*r* = 0.836) between creatinine and neopterin serum concentrations at hospital admission of patients with COVID-19 pneumonia.

Five pairs were found with an r > 0.8. However, the strong correlations between IL-6 and PCT (*r* = 0.964) and AP and gamma-GT (*r* = 0.850) were each caused by only a single extreme value. After elimination of this value, the correlations were *r* = 0.235 for IL-6/PCT and *r* = 0.358 for AP/gamma-GT (Supplementary Figure 1). The strong correlation between creatinine and urea (*r* = 0.848) was to be expected. Furthermore, neopterin and NT-proBNP (*r* = 0.833) were strongly correlated (Supplementary Figure 2). Because both parameters are elevated in patients with impaired renal function (Godai et al., 1991; Han et al., 2020), partial correlation between neopterin and NT-proBNP was calculated with creatinine as a control variable, decreasing the correlation coefficient to r = 0.590. Finally, a strong correlation (*r* = 0.836) was found between neopterin and creatinine (Figure 3).

After exclusion of all patients with creatinine >1.5 mg/dl, serum neopterin concentrations at hospital admission were no longer significantly associated with a severe course of COVID-19 (*p* = 0.403).

## 4. Discussion

### 4.1. Early prediction of severe COVID-19 in hospitalized patients is facilitated by IL-6, PCT, or CRP

The aim of this study was to identify laboratory parameters that would allow early prediction of a severe course of COVID-19 (defined as need for ventilation or death) in hospitalized patients. After evaluation of the routine laboratory parameters and experimental markers tested, three laboratory parameters turned out to be approximately equivalent: IL-6, PCT, and CRP. A serum concentration of IL-6 ≥ 52.8 pg/ml, PCT ≥ 0.11 ng/ml, or CRP ≥ 71.1 mg/L within 24 h of hospital admission could predict a severe course of COVID-19 pneumonia with a sensitivity of 92% and a specificity of 65–67%. Bacterial coinfection does not seem to confound the association of these markers with a severe outcome of COVID-19.

The role of serum IL-6 as a marker of disease progression in critically ill COVID-19 patients after admission to the intensive care unit was known from early case reports (Luo et al., 2021). Thus, IL-6 was proposed as a prognostic marker early in the pandemic (Coomes and Haghbayan, 2020). A later meta-analysis confirmed the value of IL-6 for predicting severe progression but not mortality (Liu et al., 2022). However, a recent single center study was able to show the predictive capacity of IL-6 for survival among a cohort of severe COVID-19 cases (Haroun et al., 2023). In the present study, we could show that serum IL-6 levels can also predict COVID-19 progression in a cohort of patients with a high proportion of hospitalized moderate cases. PCT and CRP were also significantly associated with severe disease progression in one of the first COVID-19 studies (Zhang et al., 2020). In later meta-analyses, both markers were shown to predict mortality as well as a severe course of the disease (Chi et al., 2022; Kumar et al., 2022). The results of these mainly retrospective studies could be confirmed in this prospective study.

Positive likelihood ratios for IL-6, PCT, and CRP were between 2.6–2.8, which represents only a small increase of the probability of a severe outcome. These markers seem more useful in negative prediction of ventilation or death in COVID-19 patients at hospital admission. With negative likelihood ratios between 0.11–0.13 the probability of a severe outcome is markedly reduced in patients below the respective threshold values. A simple score based on all three markers could help in stratification: if IL-6, PCT, and CRP on admission are all below the cut-off values established in this study, patients could be referred to standard care or outpatient care.

### 4.2. Neopterin is not an independent predictor of severe COVID-19 progression

Several studies have postulated that increased serum neopterin concentration is an independent predictor of severe COVID-19 progression, but only one has corrected for patients’ glomerular filtration rate (Bellmann-Weiler et al., 2021). Because neopterin is excreted renally (Estelberger et al., 1993) and poor renal function is an independent predictor of severe COVID-19 progression (Cheng et al., 2020), consideration of renal function is essential for this marker. In our study, elevated levels of creatinine or urea at hospital admission were also associated with severe disease progression. After exclusion of all patients with elevated creatinine levels, we could no longer demonstrate a significant association of elevated serum neopterin concentrations with ventilator requirement or death. We therefore consider the association of neopterin to severe COVID-19 progression as spurious causality.

### 4.3. Summary and limitations of the study

In this prospective observational study, we demonstrated that IL-6, PCT, and CRP were equally able to identify patients at low risk for severe COVID-19 progression already at hospital admission. All experimental parameters with a possible role in the COVID-associated cytokine storm were not suitable for predicting ventilator requirement or death in this study which was set in routine clinical care. We were able to confirm increased neopterin levels as a marker of a cellular immune response in severe COVID-19 courses but, in contrast to previous studies, consider this a spurious relationship because of the known elevation of this parameter in impaired renal function.

One limitation of the study is the low number of patients who met the primary endpoint. Since the study was planned at the beginning of the pandemic in March 2020, we assumed a significantly higher proportion of severe COVID-19 cases based on the first data from China and Italy. Furthermore, the first clinical practice guideline with recommendations for all hospitalized COVID-19 patients in Germany was not published until November 2020, toward the end of the recruitment phase (Kluge et al., 2021). Before then, there were uncertainties about the optimal therapy, so strong divergences between the study centers are possible, which may account for an unknown confounding factor. Further limitations in the transferability of the results arise from the fact that all study participants were unvaccinated and from the altered SARS-CoV-2 variants currently circulating in contrast to the April–December 2020 study period. The SARS-CoV-2 variant of concern (VOC) Alpha (B.1.1.7) was the first VOC spreading in Germany. However, it was first detected in Germany on 24 December 2020, which was after enrollment of the last patient. While it may have circulated for some time before that date, its percentage among German COVID patients during the study period is negligibly low (Dings et al., 2022). Therefore, our results are mainly representative for non-VOC strains of SARS-CoV-2.

## Data availability statement

The raw data supporting the conclusions of this article will be made available by the authors, without undue reservation.

## Ethics statement

The studies involving humans were approved by Ethics Committee of the Berlin Medical Association. The studies were conducted in accordance with the local legislation and institutional requirements. The participants provided their written informed consent to participate in this study.

## Author contributions

CZ: Conceptualization, Data curation, Formal analysis, Funding acquisition, Investigation, Methodology, Project administration, Supervision, Writing – review & editing. WW: Investigation, Methodology, Project administration, Supervision, Writing – review & editing. JK: Investigation, Writing – review & editing. UB: Investigation, Writing – review & editing. TW: Investigation, Writing – review & editing. NN: Investigation, Writing – review & editing. BF: Investigation, Writing – review & editing. RM: Investigation, Writing – review & editing. AM: Investigation, Writing – review & editing. DR: Investigation, Supervision, Writing – review & editing. MeS: Investigation, Writing – review & editing. LS: Investigation, Supervision, Writing – review & editing. FW: Investigation, Writing – review & editing. KO: Investigation, Supervision, Writing – review & editing. FK: Investigation, Writing – review & editing. MK: Investigation, Writing – review & editing. SS: Formal analysis, Validation, Writing – review & editing. MaS: Conceptualization, Data curation, Formal analysis, Funding acquisition, Investigation, Methodology, Project administration, Resources, Supervision, Validation, Visualization, Writing – original draft.

## Footnotes

^1^ https://drks.de/search/en/trial/DRKS00021591
