# Supplementary material for: Serum interleukin-6, procalcitonin, and C-reactive protein at hospital admission can identify patients at low risk for severe COVID-19 progression
Source: Front Microbiol. 2023 Oct 23;14:1256210. doi: 10.3389/fmicb.2023.1256210 (PMC10626435; doi:10.3389/fmicb.2023.1256210)
Supplement: Supplementary file 1 [file Image_1.PDF]

Supplementary Figure 1. Correlation of IL-6/PCT and AP/Gamma-GT in hospitalized patients with COVID-19 pneumonia (before and after elimination of extreme values)

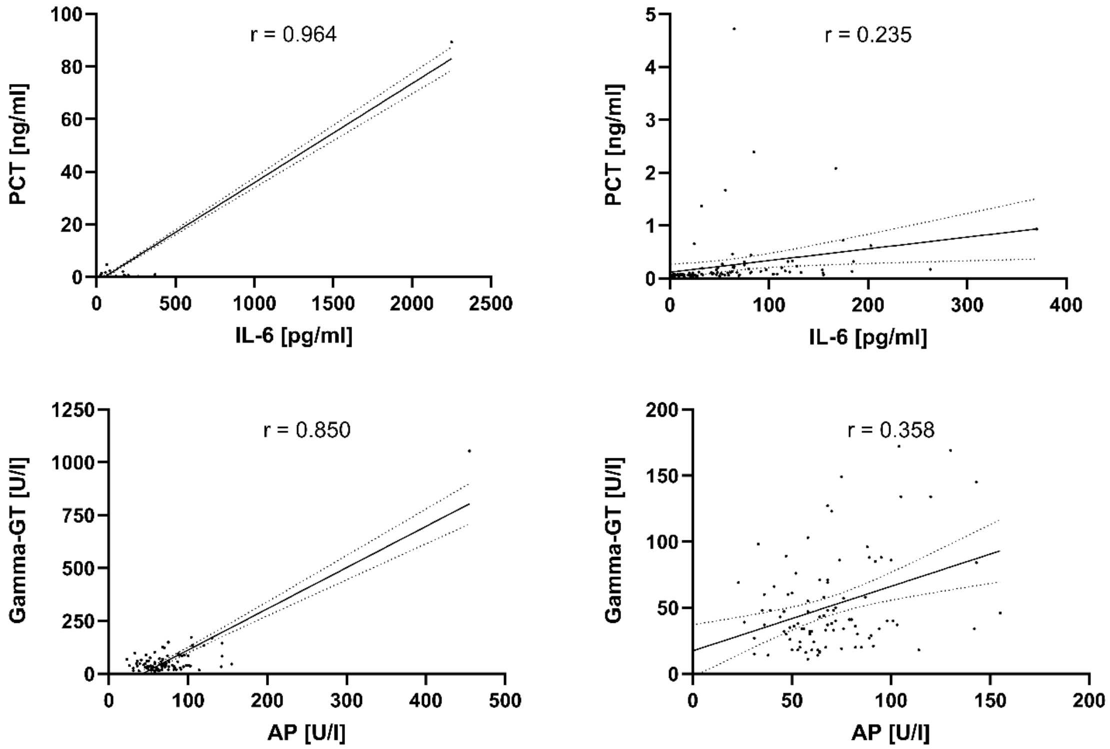

Scatter plots with linear regression line and 95% confidence interval of IL-6/PCT and AP/Gamma-GT serum concentrations at hospital admission of patients with COVID-19 pneumonia. Regression lines and correlation coefficients were calculated using all data points (left), or after elimination of one extreme value in each dataset (right).
