# Supplementary material for: Serum interleukin-6, procalcitonin, and C-reactive protein at hospital admission can identify patients at low risk for severe COVID-19 progression
Source: Front Microbiol. 2023 Oct 23;14:1256210. doi: 10.3389/fmicb.2023.1256210 (PMC10626435; doi:10.3389/fmicb.2023.1256210)
Supplement: Supplementary file 2 [file Image_2.PDF]

Supplementary Figure 2. Correlation of creatinine/urea and neopterin/NT-proBNP in hospitalized patients with COVID-19 pneumonia

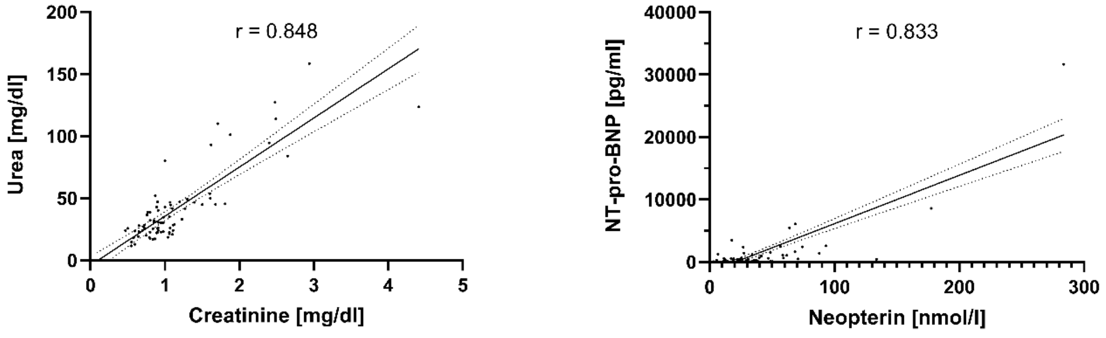

The scatter plots with linear regression line and 95% confidence interval show a strong correlation between creatinine and urea (r = 0.848), as well as neopterin and NT-proBNP (r = 0.833) serum concentrations at hospital admission of patients with COVID-19 pneumonia.
