# Supplementary material for: Serum interleukin-6, procalcitonin, and C-reactive protein at hospital admission can identify patients at low risk for severe COVID-19 progression
Source: Front Microbiol. 2023 Oct 23;14:1256210. doi: 10.3389/fmicb.2023.1256210 (PMC10626435; doi:10.3389/fmicb.2023.1256210)
Supplement: Supplementary file 3 [file Table_1.PDF]

Supplementary Table 1. Median values and significance levels of IL-6, PCT, and CRP at hospital admission of patients with COVID-19 pneumonia (after exclusion of cases with positive bacterial culture during hospital admission)

	Median (95 % confidence interval)			
**Serum marker**	**No ventilation**	**Ventilation/death**	**P-value**	**Adjusted p-value**
**IL-6**	27.1 pg/ml (20.9-47.1) [n=89]	116.9 pg/ml (52.8-185.2) [n=10]	<0.001	0.004**
**PCT**	0.07 ng/ml (0.06-0.08) [n=90]	0.2 ng/ml (0.11-0.62) [n=11]	<0.001	0.004**
**CRP**	41.5 mg/l (30-64) [n=92]	127.3 mg/l (71.2-294.6) [n=11]	<0.001	0.004**
