# Supplementary material for: Serum interleukin-6, procalcitonin, and C-reactive protein at hospital admission can identify patients at low risk for severe COVID-19 progression
Source: Front Microbiol. 2023 Oct 23;14:1256210. doi: 10.3389/fmicb.2023.1256210 (PMC10626435; doi:10.3389/fmicb.2023.1256210)
Supplement: Supplementary file 4 [file Table_2.PDF]

Supplementary Table 2. Comparison of statistical parameters for prediction of severe COVID-19 before and after exclusion of cases with positive bacterial culture during hospital admission

	Before exclusion		After exclusion	
**Serum marker**	**Pearson's r**	**AUC (95 % CI)**	**Pearson's r**	**AUC (95 % CI)**
**IL-6**	0.402	0.871 (0.774-0.967)	0.369	0.855 (0.754-0.956)
**PCT**	0.382	0.842 (0.754- 0.931)	0.367	0.841 (0.749-0.932)
**CRP**	0.376	0.841 (0.731-0.950)	0.355	0.833 (0.714-0.953)
